# Revolutionising alcohol use disorder treatment in developing countries: integrating artificial intelligence and technology-driven approaches

**DOI:** 10.3389/fpsyt.2024.1370847

**Published:** 2024-03-07

**Authors:** Akhil P. Joseph, Anithamol Babu, L T Om Prakash

**Affiliations:** ^1^ Christ University, Bangalore, Karnataka, India; ^2^ School of Social Work, Marian College Kuttikkanam Autonomous, Kuttikkanam, India; ^3^ Tata Institute of Social Sciences Guwahati Off-Campus, Jalukbari, Assam, India

**Keywords:** alcohol use disorder, mental health interventions, artificial intelligence, machine learning, technology-driven approaches, treatment

The escalating occurrence of alcohol use disorder (AUD) in developing countries, exacerbated by an inadequate strategic reorientation in treatment methodologies, demands a transition to technology-driven solutions like artificial intelligence (AI) and machine learning (ML) (1). The conventional treatment methods that follow a one-size-fits-all approach primarily centred on psychiatric models often disregard the multidimensional nature of AUD, characterised by the scarcity of resources and infrastructure (2, 3). The diverse cultural and socio-economic context of each region magnifies the intensity of this problem by resisting the in-person treatment-seeking behaviour of individuals with AUD, including inadequate support systems like community support groups, long-term psycho-social support, and family involvement in treatment and management (4, 5). This situation calls for a paradigm shift towards integrating more sophisticated, technology-driven methodologies, particularly AI and ML, which can offer holistic, adaptable, personalised, and culturally appropriate treatment approaches to accommodate the distinct requisites of individuals with AUD.

Existing research from developing countries has not yet examined the potential of AI to analyse extensive data repositories to identify problematic patterns and predictors of AUD, including the identification of underlying causes. Therefore, leveraging these technological advancements in AUD treatment and research constitutes a pragmatic shift in cost-effective AUD treatment and management, transcending conventional methodologies. This transition is critical in varied cultural contexts where conventional methods fail to access and reach. Moreover, AI and ML algorithms can sophisticate treatment strategies by customising person-centric treatment models, and continuous monitoring allows real-time adjustment to treatment plans to enhance success rates (6). This shift compels rigorous research to explore: What patterns and predictors of AUD can AI identify and conventional methods cannot? How does the manifestation of AUD range across distinct cultural settings, and how can AI contribute to a nuanced knowledge of these variations? How can AI and technology-driven knowledge be efficiently utilised to tailor treatment plans for individuals with AUD? What are the comparative outcomes of personalised treatment plans with general treatment protocols that fit all?

Exploring the potential of AI and technology-driven solutions within community-based AUD interventions in developing countries is a crucial academic endeavour. These interventions would include AI-driven social media platforms for local peer support, integration of local healthcare systems, automated text analysis for early prevention to track the digital footprints of children, educational and awareness campaigns using AI analytics, and geospatial data analysis for resource mapping, allocation, and distribution (7). However, rigorous research should also examine how AI can assist in making ethical decisions in community-based interventions, emphasising cultural sensitivity and upholding individual rights such as identifying potential biases in treatment recommendations, predicting treatment outcomes based on cultural and socioeconomic characteristics, and preventing relapse. The sustainability of such interventions might be a concern in resource-constrained developing countries. The ability of AI to enhance the impact of these interventions and cater to the unique needs and characteristics of local communities signifies a vital advancement. Utilising AI in this context has the capacity to substantially enhance community engagement in AUD treatment, proficiently linking the gap between clinical methods and the real-life experiences of individuals across diverse communities. Therefore, integrating AI could strengthen community engagement in AUD treatment by bridging clinical treatment methods and the real-life experiences of individuals in various communities. Thus, investigating the role of AI in community-based AUD interventions opens vital questions: How can AI contribute to the development of powerful, culturally appropriate strategies and ethical decision-making in community-based AUD interventions? How can AI be utilised in complex situations to develop cost-effective evidence-based strategies to ensure the optimum outcomes of AUD interventions?

Moreover, rigorously examining the efficacy of technology-driven interventions, such as telehealth, mental health applications, digital platforms for meditation, gamification strategies to manage mental health, therapies using virtual and augmented reality, AI-based chatbots, and wearable intervention monitoring devices, compared to conventional face-to-face interventions. However, while embracing the capabilities of AI and technically-driven solutions in treating and managing AUD, it is imperative to meticulously examine the feasibility and pragmatic obstacles encountered in developing countries with this paradigm shift. The uncertainty lies in the accessibility of technological resources, digital literacy and insufficient infrastructure and mechanisms for financial support. Therefore, scholarly inquiries should be entitled to develop economically viable and scalable AI and technologically-driven solutions compatible with the existing technological advancements and healthcare system in each context.

Furthermore, there is a need to cultivate technological proficiency among healthcare professionals and patients alike to ensure the efficient utilisation of these sophisticated tools through capacity-building training programmes. Moreover, it is critical to administer the need assessments and resource mapping by utilising stakeholder engagement and partnership. Collaborating with global healthcare organisations is vital for local government bodies to acquire financial backing and policy endorsements. Pilot projects are essential for evaluating the adaptability and gradual implementation of such interventions, fostering local and global applicability and cultural resonance. Thus, examining policy and governance mechanisms for integrating the capabilities of AI and other advanced technologies into the therapeutic arena demands the development and scaling up of policies. This academic pursuit is a nexus of technological innovation, healthcare policy, and cultural adaptability, thereby shaping the trajectory of AUD treatment methodologies globally.

Therefore, this letter advocates for a comprehensive, interdisciplinary research initiative that integrates clinical practices with AI and Technology-driven solutions to manage the nuances of AUD in developing countries. Sustainable interventions might enhance the current rehabilitation methods and reshape the socio-economic impact of AUD, necessitating a paradigm shift. This requires synergic effort from researchers, policymakers, and healthcare professionals to understand the complexities of AUD and develop culturally sensitive, technologically-driven interventions to prevent AUD in developing nations, marking a significant step towards progress.

## Author contributions

AJ: Conceptualization, Writing – original draft, Writing – review & editing. AB: Conceptualization, Writing – original draft, Writing – review & editing. LP: Writing – review & editing.
